# Comparative characteristics of infertile women when applying melatonin in complex preparation for assisted reproductive technologies

**DOI:** 10.25122/jml-2022-0154

**Published:** 2022-08

**Authors:** Viktoria Olexandrivna Yuzko, Olexandr Mykhailovych Yuzko, Tamara Anatoliivna Yuzko, Svitlana Hryhorivna Pryimak, Natalia Serhiivna Voloshynovych, Svitlana Ivanivna Chobaniuk

**Affiliations:** 1 Department of Obstetrics and Gynecology, Bukovynian State Medical University, Chernivtsi, Ukraine; 2 Medical Center of Infertility Treatment, Chernivtsi, Ukraine

**Keywords:** assisted reproductive technologies, melatonin, infertility, AMH – anti-mullerian hormone, ART – assisted reproductive technologies, E2 – estradiol, FSH – follicle-stimulating hormone, LH – luteinizing hormone, NAF – number of antral follicles, P – progesterone, PRL – prolactin, T4 – thyroxine, TSH – thyroid-stimulating hormone

## Abstract

A retrospective analysis of medical records of infertile patients using assisted reproductive technologies and melatonin was performed. 76 infertile women were examined. Group 1 included 33 patients who received 3 mg of melatonin two weeks before and during ovulation induction, and group 2 included 43 patients who did not take melatonin. The average age of patients in the groups did not differ. The data of gynecological and ultrasound examinations, structure and thickness of the endometrium, antral follicle count, hormone levels: anti-mullerian, follicle-stimulating, luteinizing, progesterone, estradiol, prolactin, thyrotropin, and thyroxine were evaluated. The primary infertility incidence was significantly higher in all examined patients. Patients in the first group tended to decrease ovarian reserve, recurrent loss, and unexplained infertility; in the second group, more endometriosis, tubal and male infertility factors were observed. The incidence of extragenital pathology in the examined patients did not differ as well as antral follicle count and the thickness of the endometrium. We also did not find any significant difference in the level of hormones in the blood of the examined women, except that patients taking melatonin had significantly higher levels of lutropin but lower levels of the anti-mullerian hormone in the blood.

## INTRODUCTION

The incidence of infertile marriages, according to Ukrainian and international researchers, averages from 10 to 20% [1–3]. Assisted reproductive technologies are the most effective, despite the large number of methods of conservative and surgical treatment of infertility. However, despite all the achievements of reproductive medicine, their effectiveness for pregnancy has not increased significantly and is about 40% per treatment cycle, and over the past ten years, this index has not significantly changed [4]. This is due to the large number of different factors that influence the reproductive process, and the combination of male and female factors can range from 40 to 80% [5]. Ovarian reserve in fertilization programs is an important factor because it depends on the number of oocytes obtained, their quality and viability [6]. In recent years, scientists have drawn their attention to melatonin as a possible marker of ovulatory reserve and the effectiveness of fertilization in general [7, 8].

The molecular mechanisms of melatonin are still largely unexplored. Through pituitary receptors, melatonin acts upon the secretion of pituitary hormones into the blood and, with their help – upon the reproductive system. The oocyte is surrounded by follicular fluid, *i.e*., it is a biological “window” that reflects metabolic processes [9]. It is known that the concentration of melatonin in human follicular fluid is higher than in serum. Active absorption of melatonin by follicles from blood and own synthesis works [10–12].

The study of the anamnesis peculiarities, ovulatory reserve, and hormonal status in infertile patients undergoing assisted reproductive technologies with melatonin has not been sufficiently studied. It is known from very few literature sources [13] that the incidence of pregnancy in patients who received melatonin during the month in preparation for fertilization was significantly higher.

## MATERIAL AND METHODS

We examined 76 infertile women. Group 1 included 33 patients who took 3 mg of Vita-melatonin produced by the Kyiv Vitamin Plant at the same time before bedtime, two weeks before and during ovulation induction. Group 2 included 43 patients who did not take melatonin. The study did not include women who worked night shifts. We studied outpatient records, as well as gynecological and ultrasound examination data and hormone levels in the blood. The ultrasound examination of the lesser pelvis organs was performed on the Mindray DC-80 X-Insight device with a transvaginal sensor. The structure and thickness of the endometrium were assessed, and the number of antral follicles (NAF) ranging in size from 2 to 10 mm in each ovary was counted. Serum levels of hormones were determined in all patients: anti-mullerian, follicle-stimulating, luteinizing, estradiol, prolactin, progesterone, thyroid-stimulating hormone, and thyroxine by immunofluorescence. For this purpose, standard kits of the DELFIA system and IFA method “STAT-GRAFIX” 20/09 were used. To process the obtained results, the method of variation statistics was used with the calculation of the arithmetic mean (M), the average error of the mean (m), and the probability (p). The reliability of parametric values was evaluated by the probability of Student's criterion.

## RESULTS

The mean age of women in Group 1 (who took melatonin) was 33.12±8.18 years, and in group 2 (who did not take melatonin) – 30.95±7.07 years (p>0.05), i. e. according to this parameter, they were equal.

According to Table 1, the frequency of primary infertility in group 1 was 72.72±7.75%, while in group 2 – 62.79±7.35% (p>0.05), with no difference. The frequency of secondary infertility in group 1 (27.27±7.75%) compared with group 2 (37.21±7.35%) also had no significant difference (p>0.05). At the same time, in all the patients examined, the incidence of primary infertility was significantly higher than that of secondary infertility (2.7 times in group 1 and 1.7 times in group 2).

**Table 1:** The incidence of primary and secondary infertility in the women examined.

Infertility, %	Group 1, n=33	Group 2, n=43
**Primary**	72.72±7.75	62.79±7.35
**Secondary**	27.27±7.75	37.21±7.35

If we consider the factors of infertility that led to the use of *in vitro* fertilization in the examined patients (Table 2), it should be noted that in group 2, endometriosis was 1.8 times more common, tubal factor – 1.75 times, male factor – 1.5 times, while in group 1 common infertility and infertility of unclear genesis were 2.6 times more common, reduced ovarian reserve – 3.9 times, but the difference was insignificant (p>0.05).

**Table 2:** Reasons for *in vitro* fertilization use in infertile patients, %, M±m

Reasons for infertility	Group 1, n=33	Group 2, n=43
**Endometriosis**	9.09±5.03	16.28±5.74
**Tubal factor**	21.21±7.11	37.21±7.45
**Male factor**	15.15±6.35	23.26±6.53
**Recurrent pregnancy loss**	6.06±4.14	2.33±2.24
**Polycystic ovary syndrome**	12.12±5.75	11.63±4.94
**Unexplained infertility**	12.12±5.73	4.65±3.23
**Diminishing ovarian reserve**	18.18±6.74	4.65±3.25
**Sperm donation**	3.03±3.01	-
**Uterine factor**	3.03±3.02	-

According to Table 3, the number of extragenital pathologies, in general, did not differ significantly between groups, except for goiter in group 1, which occurred 2.2 times more often, and pyelonephritis – 3.3 times, although the difference was insignificant (p>0.05).

**Table 3:** Extragenital disease in the examined patients, %, M±m.

Indexes	Group 1, n=33	Group 2, n=43
**Pathology of the thyroid gland**		
Autoimmune thyroiditis	6.06±4.13	-
Hypothyroidism	3.03±3.03	-
Goiter	15.15±6.31	6.98±3.92
Hyperthyroidism	3.03±3.01	-
**Urinary System Pathologies**		
Pyelonephritis	15.15±6.33	4.65±3.24
Cystitis	3.03±3.02	-
**Obesity**	15.15±6.32	2.33±2.24
**Cardiovascular pathology**	6.06±4.13	9.30±4.54
**Gastrointestinal pathology**	6.06±4.12	4.65±3.23
**Cholecystitis**	3.03±3.01	4.65±3.24
**Varicose veins**	3.03±3.02	2.33±2.25

Evaluating the results of ovarian ultrasound and endometrial thickness, it should be noted (Table 4) that NAF in the right ovary in group 1 (6.92±1.05) and group 2 (7.74±0.91) did not differ. A similar situation was with NAF in the left ovary. The thickness of the endometrium also did not differ between patients, respectively, 7.13±1.06 mm and 6.92±0.92 mm.

**Table 4:** Ultrasound examination data (M±m).

Indexes	Group 1, n=33	Group 2, n=43
The number of antral follicles in the ovary		
**Right**	6.92±1.05	7.74±0.91
**Left**	7.24±1.06	7.93±0.91
Endometrium, mm	7.13±1.06	6.92±0.92

Regarding the studied hormone levels (Table 5), we found no differences in the content of FSH, E2, PRL, P, TSH, or T4 in blood. The level of LH in the blood of group 1 (19.6+0.15 mIU/ml) was significantly (p<0.001) higher than the same index (8.8+0.10 mIU/ml) in group 2. However, the level of AMG (3.3+0.29 ng/ml) in group 1 was probably (p<0.001) lower than this index (8.4+0.23 ng/ml) in group 2 accordingly.

**Table 5:** The level of hormones in women's blood.

Indexes	Group 1, n/M±m	Group 2, n/M±m
FSH, mIU/ml	25/7.54±0.56	43/8.15±0.63
LH, mIU/ml	25/8.64±0.15	40/8.83±0.10
E2, pg/ml	22/66.21±7.31	36/52.71±4.12
PRL, ng/ml	32/17.94±1.49	41/18.83±1.48
P, ng/ml	23/0.62±0.052	37/0.41±0.012
AMH, ng/ml	32/3.43±0.29	42/8.44±0.23*
TSH, nmol/l	10/1.84±0.16	12/1/52±0.11
T4, nmol/l	8/2.52±0.40	8/2.53±0.24

* – significant difference between groups (p<0.001).

## DISCUSSION

Infertile marriage has been and remains a serious medical and social issue. It should be noted that the incidence of this pathology varies widely, from 0.2 to 20%, and averages 10–20%, according to Khazhilenko K. [1] and Yuzko OM *et al*. [2]. In addition, cases of male infertility are almost two times fewer. Along with the low birth rate, the problem acquires national significance [14, 15]. Accordingly, its solution goes beyond medicine.

Despite the wide range of conservative and surgical treatment methods, nobody has been able to find the only effective one. This is clear because there are about 30 causes of male and female infertility according to the works of Bulavenko OV *et al*. [5] and Khmil SV. *et al*. [16]. The most effective treatment method for male and female infertility in recent decades is assisted reproductive technologies. [3, 17, 18]. They are constantly being improved, and their efficiency increased [19, 20]. At the same time, as shown by several studies, their effectiveness depends on the age of patients, medical history, treatment, especially surgery, existing extragenital diseases etc [21–24]. The condition of the uterus and endometrium is equally important, as mentioned by Drozdovska YuB [25]. Two components are crucial: ovarian reserve and oocyte quality [6, 26, 27]. Ayzyatulova EM indicates that ovarian reserve is directly related to a woman's age, ovarian condition, hormonal balance, and the number of antral follicles [28]. Markers of the ovulatory reserve are well known today. The main ones are NAF (number of antral follicles determined by ultrasound) and AMG (anti-mullerian hormone level in blood) with E2 (estradiol), P (progesterone), FSH (follitropin), and LH (lutropin) [29, 30]. Nevertheless, the search for ovulatory reserve markers continues. In recent years, melatonin has attracted the attention of scientists as a possible marker of ovulatory reserve and the effectiveness of fertilization in general, which is confirmed by Orlova VV *et al*. [7] and Tamura H. *et al*. [8].

The molecular mechanisms of melatonin action in the pineal gland have not been fully studied. It is assumed that melatonin acts upon the secretion of hormones into the blood and with their help upon the reproductive system through pituitary receptors. Tamura H. *et al*. emphasized that regarding oocytes surrounded by follicular fluid, melatonin acted as a biological “window” associated with the metabolic and hormonal characteristics of oocytes and granulosa cells [9]. Fumio Otsuka [10], Qing He *et al*. [11], and Jamilian M. *et al*. [12] draw attention that the level of melatonin in human follicular fluid is much higher than in serum, which takes place due to the active absorption of melatonin by follicles from serum and its synthesis by the follicles themselves.

Study data presented by Yuzko VO [13] show that the incidence of pregnancy in patients who took melatonin during the month in preparation for fertilization was 60.6±8.25%, which is probably higher than in patients who did not take melatonin (45.0±7.62%, p<0.05).

Due to the above, it is of great interest to study the medical history, ovulatory reserve, and hormonal status in infertile patients when performing assisted reproductive technologies using melatonin described in works by Orlova VV *et al*. [31–33] and Svyrydova NK *et al*. [34].

This work presents the study results of 33 patients who took melatonin within one month before the puncture of the follicles in fertilization programs and 43 patients who did not take melatonin.

The mean age of women who took and did not take melatonin did not differ. The incidence of primary and secondary infertility in groups 1 and 2 also did not differ. At the same time, the incidence of primary infertility in patients of both groups examined was generally much higher than the secondary one.

If we consider the factors of infertility that led to the use of *in vitro* fertilization in the examined patients, it should be noted that endometriosis, tubular factor, and male factor in women who did not take melatonin were more common, while in the group using melatonin there were miscarriages and infertility of unclear genesis, reduced ovarian reserve, but the difference was small and inaccurate. The number of concomitant extragenital pathology, in general, did not differ significantly between the groups, except for goiter in group 1, which was 2.2 times more common, and pyelonephritis – 3.3 times, although the difference was inaccurate and insignificant.

Evaluating the results of ultrasound examination of the ovaries, it should be noted that NAF in the ovaries of patients did not differ between groups, as well as the thickness of the endometrium.

We did not find any significant difference in the content of FSH, E2, PRL, P, TSH, and T4. The level of LH in the blood of patients in group 1 was significantly (p<0.001) higher than in group 2. At the same time, the AMG level in group 1 was lower than in group 2 (p<0.001).

Summing up the discussion, it should be noted that the examined patients who took and did not take melatonin in preparation for ART did not differ in age, medical history, extragenital pathology, ultrasound examination data, and hormone levels in the blood, *i.e*., they were comparable.

## CONCLUSION

The presented literature data and the results of our study show that the use of melatonin in ART programs increases their effectiveness. Analysis of the medical records of examined patients showed that infertile patients who did not receive this preparation in such programs did not differ in age, the incidence of infertility, factors that led to infertility, concomitant extragenital pathology, ovarian reserve, and reproductive hormone levels. That is, in our study, they were equal.
